# Longitudinal CT number characterization of a novel upright CT for proton therapy planning

**DOI:** 10.1002/acm2.70685

**Published:** 2026-07-07

**Authors:** Yuhao Yan, Jordan M. Slagowski, Jessica R. Miller, John W. Hayes, Minglei Kang, Carson A. Hoffman, Carri K. Glide‐Hurst

**Affiliations:** ^1^ Department of Radiation Medicine University of Wisconsin‐Madison Madison Wisconsin USA; ^2^ Department of Medical Physics University of Wisconsin‐Madison Madison Wisconsin USA; ^3^ Leo Cancer Care Middleton Wisconsin USA

**Keywords:** imaging for particle therapy, upright CT, upright radiotherapy

## Abstract

**Background**: The combination of upright CT and patient positioner for particle therapy presents potential for high precision image‐guided radiation therapy and online adaptive radiation therapy, where reliable CT performance is essential.

**Purpose**: To evaluate the longitudinal stability of upright CT for proton radiation therapy, benchmark against standard of care conventional CT, and establish the feasibility of a simplified phantom configuration for efficient routine quality assurance (QA).

**Methods**: Following consensus guidelines, a calibration phantom with tissue mimicking inserts located at variable phantom positions was scanned on an upright CT (120 kV, 250 mAs) 14 times over 7 months. CT number intersession repeatability was assessed via standard deviation (SD). To assess size dependency due to beam hardening, body and head phantom measurements were evaluated. A Hounsfield look‐up table (HLUT) for stopping power ratio (SPR) estimation was derived for proton dose calculations. The phantom was scanned on a conventional CT (120 kV, 250 mAs) for benchmarking. An anthropomorphic phantom (ATOM) was scanned on upright and conventional CT. Proton plans were developed in the phantom for prostate and spine stereotactic body radiation therapy on upright CT using pencil beam scanning techniques, robust optimization (3–5 mm setup, 3.5% range uncertainties), and Monte Carlo dose calculation. Dose was computed on co‐registered conventional CT datasets. Dose agreement on upright and conventional CT was assessed. To develop an efficient approach for routine QA, a simplified phantom configuration (1 scan with 4 bone inserts) was scanned on upright CT over 8 months (15 imaging sessions, 5 images/session). Inter‐ and intra‐session repeatability were assessed. The Wilcoxon signed‐rank tests were used to test for significant differences between head/body and consensus/simplified phantom configurations.

**Results**: Using consensus phantom configurations, upright CT demonstrated excellent longitudinal CT number stability with minimal inter‐session SD ≤4.9 HU. System upgrades and recalibration introduced marginal offsets (difference in CT numbers ∆HU ≤14.4 HU). Size dependency of beam hardening was identified with statistically significant (*p* <0.05) differences in upright CT numbers (maximum ∆HU = 136 HU in cortical bone), leading to |∆SPR| up to 0.079 comparing body and head phantom results. Comparing proton dose calculated on upright and conventional CT using corresponding consensus‐derived HLUTs, for both spine and prostate plans, local dose differences were found at distal end of beam path due to SPR differences with minimal target coverage differences <0.3% and gamma pass rates >99% at 1 mm/1%. Using a simplified phantom configuration, longitudinal upright CT number stability was also excellent (inter‐session SD ≤3.2 HU and intra‐session SD≤1.6 HU) although when comparing results of the consensus versus simplified phantom configurations, upright CT numbers were substantially different (*p* <0.05, maximum ∆HU = −76 HU in cortical bone) yielding |∆SPR| up to 0.044.

**Conclusions**: Upright CT demonstrated excellent longitudinal CT number stability as required for treatment planning and as a step toward adaptive proton therapy. Differences were noted between body and head phantom results, suggesting value in size‐specific calibration protocols. The feasibility of using a simplified phantom configuration was demonstrated to support efficient QA to monitor machine stability. Future work includes extending the investigation to multi‐institution validation.

## 1 INTRODUCTION

With the introduction of novel upright patient positioning systems,^1^, ^2^, ^3^ there has been an increasing interest in delivering radiation therapy (RT)—particularly particle therapy—with patients in the upright orientation (e.g., seated, perched) by rotating patients relative to a fixed beamline. By eliminating the need of a large rotating gantry, upright particle therapy allows small footprint of the treatment room and easier construction and maintenance of the delivery system, reducing the cost and offering potential improvements to the accessibility of particle therapy.^1^, ^2^ In addition, when patients are positioned upright, potential anatomical benefits may be introduced, such as increasing lung volume, reducing tumor motion in the lung,^3^, ^4^ stabilizing anatomy in the pelvis,^5^ and mitigating dyspnea or other medical conditions that might be introduced lying down for head and neck cancer patients,^6^ potentially leading to better normal tissue sparing and patient comfort.

To support upright RT, imaging of patients in the upright orientation is necessary for simulation, treatment planning, and daily image guidance. Novel imaging systems have been introduced where the CT bore translates vertically relative to the patient positioned in the upright orientation,^7^, ^8^, ^9^ contrary to conventional standard of care CT scanners where the gantry is fixed while the patient is positioned in the recumbent orientation (e.g., supine, prone) and translated by the couch. One distinct benefit of integrated vertical CTs at the treatment isocenter is that uncertainty is reduced as the patient does not move between imaging and treatment isocenters, thereby minimizing anatomical variations and uncertainties.^1^, ^10^ Isocentric upright CT enables rapid acquisition of high quality CT images, which is an essential step to support future online adaptive radiation therapy (ART) implementations.

To enable proton dose calculation, CT numbers need to be converted to stopping power ratio (SPR). One of the common approaches is to establish a Hounsfield look‐up table (HLUT) between CT number and SPR via stoichiometric calibration,^11^ where tissue‐equivalent materials are scanned to relate CT numbers to their known chemical composition, which is further used to estimate SPR via the Bethe equation. Alternatively, empirical calibration can be adopted to directly measure the SPR of the tissue‐mimicking inserts.^12^ Longitudinal stability of CT numbers is critical to enable accurate and consistent dose calculations on planning and daily CT images while using the same HLUT,^13^, ^14^ as it has been reported that longitudinal variation in CT number translates to variations in SPR of similar scale.^15^

Recently, one of the first clinical installations of a novel isocentric upright CT scanner coupled with a 6 degree‐of‐freedom upright patient positioner was performed for integration with a fixed proton therapy beam line. Currently there is no published longitudinal data on the upright CT performance including CT number stability, which is critical to ensure consistent and reliable SPR conversion for safe clinical implementation. Toward this need, this work sought to quantify the longitudinal repeatability of upright CT number over 8 months of operation. In preparation for the emerging upright proton RT programs, considerations were given to derive the HLUT between CT number and SPR following established consensus guidelines.^16^ Dose agreement with standard of care conventional CT was assessed. In addition, the suitability of using a simplified phantom configuration to monitor CT number stability while shortening routine quality assurance (QA) was assessed, including evaluating CT number stability, CT number agreement, and HLUT agreement with results obtained from the comprehensive consensus measurement technique.

## 2 METHODS

### 2.1 Upright CT specifications

**FIGURE 1:**
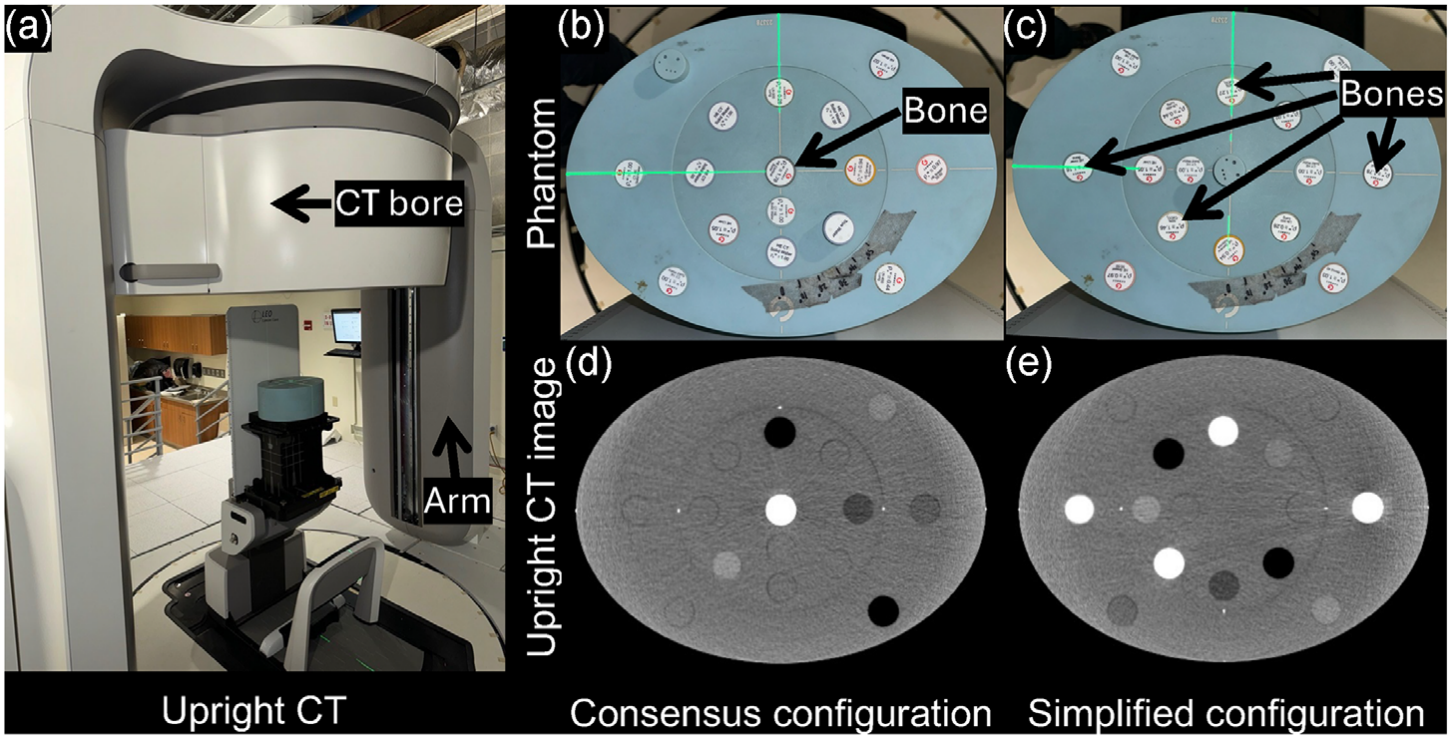
Demonstration of (a) the upright CT and the calibration phantom setup, along with different insert configurations and corresponding upright CT images including (b, d) consensus (Peters et al., 2023) and (c, e) simplified configuration. Note that reconstructions for upright CT demonstrated slightly brighter areas in the long‐axis periphery of the oval‐shaped phantom, which were not observed in the conventional CT images.

The Marie system (Leo Cancer Care, Middleton, WI) consists of an upright patient positioner coupled with an upright CT and was installed for pre‐clinical testing (Serial Number 6, Figure 1a). The CT system is comprised of three major components: the pillars that support the arms, the arms that support the CT bore with associated translational hardware and execute the tilt of ± 5°, ± 10° and ± 15°, and the 85 cm diameter CT bore that translates vertically within a range of 130 cm for our configuration.^17^ The CT is coupled with a 6 degree‐of‐freedom patient positioning system (chair). The maximum reconstruction field‐of‐view (FOV) is 62.3 cm.^17^ The CT is designed to operate within 80–140 kV tube voltage, while only 120 kV was calibrated for clinical use. Other parameters include pitch of 0.5–1.5 (9.65–28.95 mm/s), rotation time of 1.0 s, and tube current up to 250 mA.

During the 8‐month course of study, it is estimated that the upright CT was utilized ∼2 days per week for research activities (e.g., tube warm‐up and air calibration, Catphan/ACR phantom scans, anthropomorphic phantom scanning, manufacturer evaluations) for ∼5–8 h per day outside of the stability monitoring sessions.

### 2.2 HLUT specifications following consensus guidelines

To characterize the upright CT for proton dose calculations, an Advanced Electron Density Phantom (Sun Nuclear, Melbourne, FL) was used, which included tissue‐mimicking inserts (lung, adipose, soft tissue, and bone) with known density and chemical compositions. The CT number measurements and HLUT specifications followed established consensus guidelines as described by Peters *et al* ^16^ via the European Particle Therapy Network. The consensus guidelines outline key steps including acquiring 14 CT scans following unique insert arrangements to minimize beam hardening and calculating the HLUT following the Bethe equation.

For each imaging session, 14 scans were acquired following the consensus guidelines including: (1) five scans to measure insert CT numbers with the body phantom, where one initial scan was acquired with all non‐dense inserts placed in the phantom, and four additional scans were acquired, each with the central insert replaced with one of the four bone inserts (nominal mass density 1.21–1.93 g/cm^3^) to reduce uncertainty from beam hardening, (2) four scans using the body phantom to evaluate the potential impact of beam hardening due to insert location for dense inserts, each with one periphery insert at a fixed location replaced with one of the four bone inserts, and (3) five scans to measure insert CT numbers with the head phantom following the same procedure as (1). An example of phantom configuration is shown in Figure 1, with detailed insert arrangement listed in supplement document Figure S1A. All scans were performed using an identical protocol of 120 kVp, 250 mA, pitch of 1.0, 1 s per rotation, ∼1 mm^2^ in‐plane resolution, 500 mm^2^ FOV, 512^2^ in‐plane matrix, 2 mm slice thickness, and a vendor‐supplied RamLak reconstruction kernel using filtered backprojection. Mean CT numbers were automatically extracted using a Python algorithm developed based on Pylinac,^18^ within a cylindrical region of interest (ROI, 8 mm radius and 10 slices/20 mm height) automatically identified in the center of each insert.

To assess the longitudinal stability of the CT numbers with the best accuracy and minimal beam hardening uncertainties, 14 imaging sessions were performed following the consensus guidelines over a span of 7 months (Jan‐July 2025). Intersession repeatability was assessed by calculating the standard deviation (SD) and coefficient of variation (CV; i.e., the SD of all measurements for each phantom insert divided by their mean) of the 14 sessions using the body phantom configuration. During the course of the study, the upright CT underwent software upgrades twice (March and May of 2025), with a recalibration of the water CT number using an ACR 464 phantom (Sun Nuclear) during the first software upgrade. Absolute and percentage differences for each timepoint were calculated referring to the initial timepoint as the baseline.

The SPR HLUT was derived for proton dose calculation based on the CT scans acquired at the imaging session in which CT scans of an anthropomorphic phantom were also performed (see below), following an algorithm and freely‐available software provided by the consensus guidelines.^16^ Key details of the algorithm are summarized below as described in the consensus guidelines.^16^ SPR of each insert was computed following the Bethe equation in combination with mean excitation energies from ICRU‐49.^19^ Tabulated human tissues^20^ were included to improve the calibration stability where CT number estimation^21^ and SPR calculation were performed. After grouping by four tissue types (lung, adipose, soft tissue, and bone), piecewise linear regression was performed between CT numbers and SPRs to derive the HLUT.

Per consensus guidelines, body and head‐specific HLUTs were derived individually following the above methodology. CT numbers and HLUTs acquired with the body and head phantoms were compared to assess the potential size‐dependency of beam hardening and the need of size‐specific HLUT. Bland‐Altman plots were generated to depict the differences between CT numbers acquired with the body and head phantoms. For each insert, CT numbers were firstly averaged across all 14 sessions, then the difference for each insert between the two phantom acquisitions (HU_head_—HU_body_) was plotted against their mean ((HU_head_ + HU_body_)/2). To test for statistically significant differences between CT number quantification using the body and head phantom, Wilcoxon signed‐rank tests were performed for each insert using the paired data from the 14 sessions. *P*‐values were adjusted by applying Bonferroni correction to account for multiple comparisons across the 12 inserts with *p* <0.05 considered statistically significant. Additionally, CT numbers of the dense bone inserts measured in the center or the periphery locations in the body phantoms were compared to further assess location dependency.

### 2.3 Validation against conventional CT

As a comparator to the HLUT specification using standard of care technology (e.g., conventional CT), a single imaging session following the consensus guidelines was performed on a Siemens SOMATOM Definition Edge (Siemens Healthineers, Erlangen, Germany) using a matched imaging protocol (120 kVp, 250 mA) with the exception of the reconstruction kernel (Br38s, a vendor‐specific standard kernel for body imaging). CT numbers were extracted, HLUT was derived, and differences between the body and head phantom CT results acquired on the conventional CT were assessed following the methodologies described above.

The clinical equivalence of proton dose calculations between the upright and conventional CTs was evaluated. An anthropomorphic phantom (CIRS ATOM Adult Male Model 701, Sun Nuclear) was scanned on the upright CT (120 kVp, 500 mm^2^ FOV). Tumor‐mimicking targets were delineated to represent the spine and prostate/seminal vesicles. Proton treatment plans were developed in the RayStation treatment planning system (RaySearch Laboratories, v2024A SP3, Stockholm, Sweden) for each target using pencil beam scanning techniques and a Hitachi ProBeat beam model. The spine plan included three beams (posterior, left posterior‐oblique (50°) and right posterior‐oblique (50°)) prescribed to 9 Gy (RBE)/fx for 3 fractions to target D_95_, which was robustly optimized using 3 mm setup uncertainty and 3.5% range uncertainty following standard clinical planning guidelines. The prostate plan included two opposing beams (right lateral and left lateral) prescribed to 8 Gy (RBE)/fx for 5 fractions to target D_98_, which was robustly optimized using 5 mm setup uncertainty and 3.5% range uncertainty. Dose was calculated using the body HLUT derived from measurements on the same day as the anthropomorphic scan. The Monte Carlo v5.6 dose engine was used with 0.5% statistical uncertainty for all final dose calculations. The ATOM anthropomorphic phantom was scanned on the conventional CT using a matched protocol with the exception of the vendor‐specific reconstruction kernel. The conventional CT dataset was rigidly registered to the upright CT dataset, and the plan was recalculated on the conventional CT dataset using the corresponding body HLUT. To evaluate dosimetric differences, target coverage was compared, and local 3D Gamma analysis was performed at 1 mm/1% criterion with 10% dose threshold between the upright CT and conventional CT dose.

### 2.4 Longitudinal dosimetric sensitivity analysis

To put the CT number deviation from the software upgrade and recalibration into clinical context and investigate the dosimetric impact of the differences, dose calculations with identical plans were performed on the same anthropomorphic CT images for the prostate and spine with HLUTs from January 9, 2025 (first available timepoint and before the first software upgrade and CT number recalibration) and June 23, 2025 (after the second software upgrade, same day as anthropomorphic phantom scan) and dose differences were assessed. Details are described in the supplement document.

### 2.5 Simplified phantom configuration for routine QA

In addition to the consensus measurements using multiple insert configurations for rigorous HLUT characterization, an alternate phantom configuration was assessed using a single insert arrangement to simplify the acquisition process to consider for routine QA purposes.^22^ As the upright scanner is expected to support online ART procedures, establishing an efficient means of performing routine CT QA over a wide array of tissue‐mimicking inserts that densely samples the physiological range is advantageous for comprehensive monitoring of CT number stability. Thus, a simplified phantom configuration (termed “simplified” in this work) was scanned with all four bone inserts simultaneously placed in the body phantom (Figure 1c,e) such that CT numbers of all inserts could be extracted using a single scan, in contrast to the consensus configuration where five scans were needed to measure CT numbers of all inserts. The simplified approach enables efficient tracking of CT number stability of all tissue‐mimicking inserts and deviations in these data could be used to trigger a full consensus configuration data acquisition and subsequent HLUT update. All insert arrangements are shown in detail in supplement document Figure S1B.

The simplified configuration was scanned during 15 sessions over a span of 8 months (Dec 2024‐July 2025) with five repeat acquisitions performed consecutively without repositioning the phantom at each of the 15 imaging sessions. At each imaging timepoint, the mean and SD across the five repeat acquisitions were calculated, and the intrasession CV was calculated. The range of intrasession CT number repeatability (SD and CV) from the 15 sessions was reported. The CV describing the intersession repeatability was calculated across sessions 1–15 using the overall mean and SD across the 15 sessions. While assessment of the CT number stability within the simplified configuration is the primary goal, to understand the impact of the insert configurations on CT number measurements, CT numbers acquired with the simplified configuration were compared with the consensus results obtained at 14 matched timepoints by calculating absolute and percentage differences. To test for statistically significant differences between CT number quantification following the consensus and simplified configurations, Wilcoxon signed‐rank tests were performed using the data from the 14 matched sessions. *P*‐values were adjusted applying Bonferroni correction for multiple tests with *p* <0.05 considered statistically significant.

Despite not being intended for clinical HLUT specifications, to understand the impact of increased uncertainties of beam hardening and scatter compared to the consensus guidelines, the HLUT was derived for the simplified configuration following the same methods for the consensus configuration and compared with the consensus results. A single session following the simplified configuration was also acquired on the same conventional CT using an identical imaging protocol (120 kVp, 250 mA, Br38s). CT numbers were extracted, and HLUT was derived and compared to the consensus configuration.

## 3 RESULTS

**FIGURE 2:**
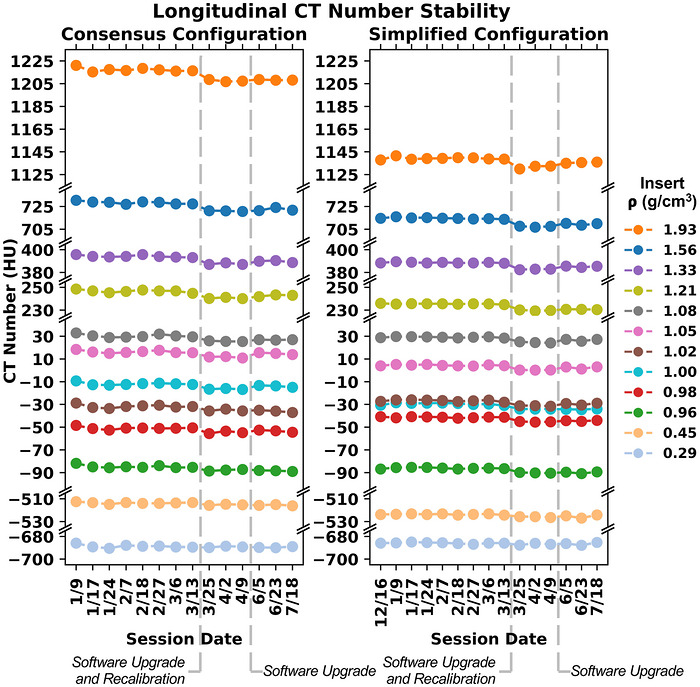
Longitudinal stability of upright CT number acquired following different phantom configurations using the body phantom (Dec 2024–July 2025).

Figure 2 summarizes the longitudinal stability of upright CT numbers extracted from the scans acquired with the consensus or simplified configurations using the body‐sized phantom. Software upgrades and recalibration timepoints are noted. Supplement Table S1 summarizes longitudinal statistics for each insert scanned at upright CT with different phantom configurations. Overall, excellent repeatability of upright CT numbers was observed for all inserts with intersession SD ≤4.9 HU when scanned following the consensus configuration. Similarly, for the simplified configuration, excellent repeatability was observed with intersession SD ≤3.2 HU and intrasession SD ≤1.6 HU. For both consensus and simplified configurations, inter‐ and intra‐session CVs were within 8% for all inserts except for a few outliers due to small mean values (water, HE brain, HE liver).

Supplement Table S2 summarizes upright CT number deviations of each timepoint from the first data acquisition baseline following the consensus configuration with the body‐sized phantom, grouped by timelines defined by system upgrades. Before the 1st upgrade, among all inserts, the largest mean ∆HU across the 7 timepoints relative to the baseline was cortical bone ∆HU = −4.3 HU. Individual measurements showed ∆HU ranging from −5.8 to 0.0 HU across all timepoints and all inserts. After the first upgrade, the largest mean ∆HU relative to the baseline increased to −13.6 HU for cortical bone. Individual measurements showed larger ∆HU, ranging from −14.4 to −2.2 HU. After the second upgrade, the largest mean ∆HU was −12.9 HU (cortical bone) with individual measurements ∆HU ranging from −13.1 to −2.5 HU, comparable to those after the first upgrade. To assess the longitudinal CT number variability independent of software upgrade and CT number recalibration, CT numbers were grouped by software version and calibration condition at the time of acquisition and compared separately (*n* = 8, 3, and 3). Overall, the differences between any two measurements within each group were within 5.8 HU across all inserts, suggesting minimal longitudinal CT number variation within the same software version and calibration condition.

Supplement Figure S2 demonstrates the variations in CT numbers between January 9 and June 23 and the resultant differences in HLUTs. As shown in Fig S2A, CT numbers measured on January 9 were systematically higher than June 23 with minor ∆HU ∼5 HU for most inserts but 13.1 HU for cortical bone (*ρ* = 1.93 g/cm3). Fig S2B demonstrates excellent agreement of the two HLUTs, with Fig S2C suggesting maximum ∆SPR = 0.026 occurred at −142 HU. Supplement Figure S3 summarizes comparison of dose calculation using HLUTs from different CT calibrations for the spine and prostate plans. Dose difference maps demonstrated minimal differences for both plans. The DVHs showed excellent agreement with negligible difference in target coverage (∆D_95 _= 0.0% for the spine plan and ∆D_98 _= 0.0% for the prostate plan). Gamma test pass rates were 100.0% at 1 mm/1% for both plans.

**FIGURE 3:**
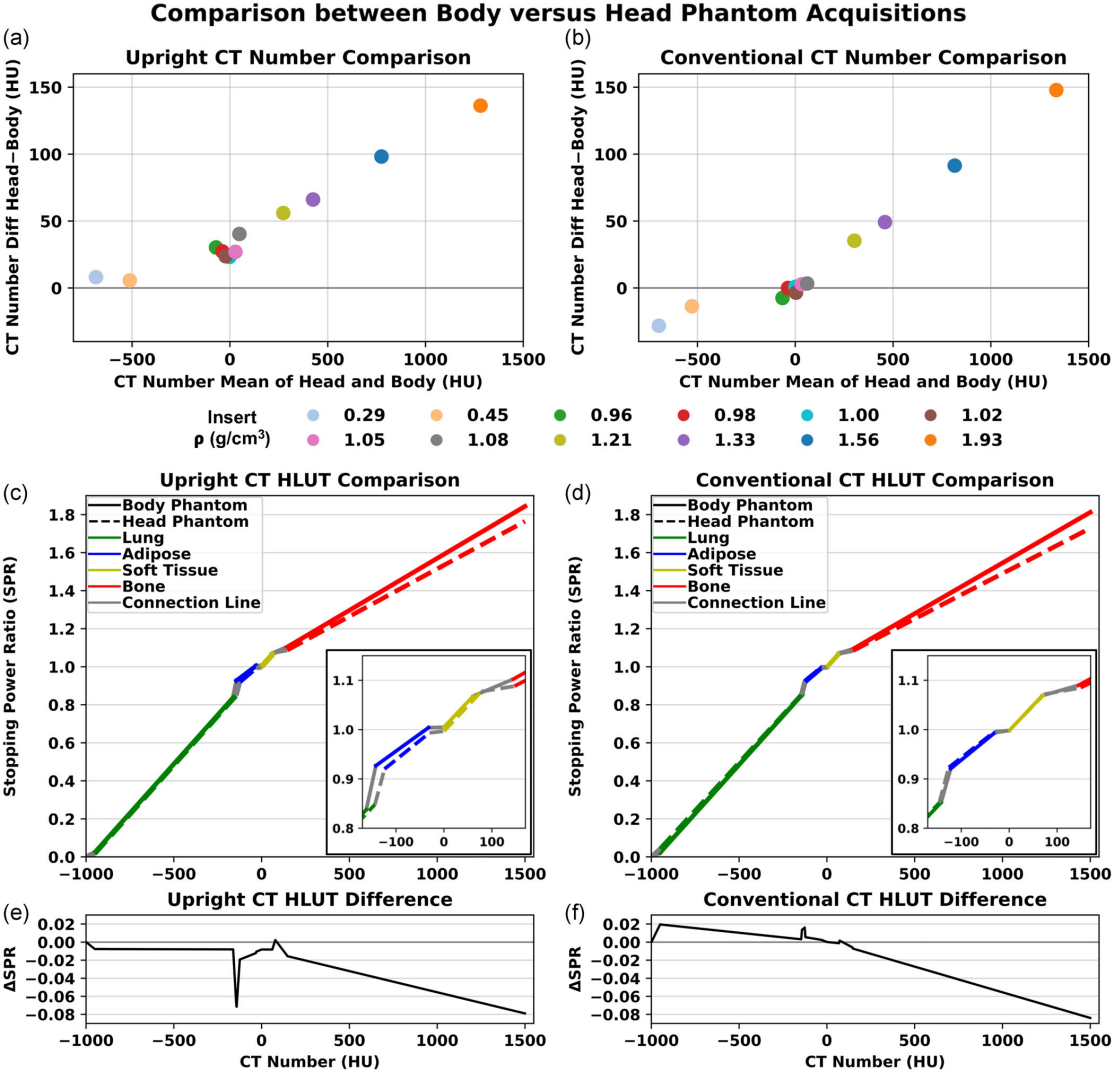
Comparison of acquisitions using the body versus head phantom following the consensus configuration to assess the potential impact of phantom size, including (a) Bland‐Altman plot of CT numbers on the upright CT and (b) the conventional CT, (c) Hounsfield look‐up tables (HLUTs) on the upright CT and (d) the conventional CT, and (e) HLUT differences on the upright CT and (f) the conventional CT. A zoomed‐in view highlighting adipose and soft tissue is shown in the bottom right for HLUT.

To assess the potential impact of beam hardening and scatter due to phantom size, Figure 3a,b demonstrates Bland‐Altman plots comparing CT numbers acquired with the body versus head phantom following the consensus configuration on the upright and conventional CT, respectively. For the upright CT, CT numbers were generally higher using the head phantom for adipose, soft tissue and bone inserts, ranging from ∆HU = 23 HU (water, *ρ* = 1 g/cm^3^) to ∆HU = 136 HU (cortical bone, *ρ* = 1.93 g/cm^3^), while for the two lung inserts, the differences in HU were negligible (≤8 HU (*ρ* = 0.29–0.45 g/cm^3^)). Wilcoxon signed‐rank test yielded statistically significant differences (*p* <0.05) between upright CT number quantifications using the body versus head phantom for all inserts. By comparison, for the conventional CT, CT numbers agreed well between the two phantom sizes for soft tissue/adipose inserts (|∆HU|≤8 HU for *ρ* = 0.96–1.08 g/cm^3^) while larger discrepancies were observed for lung (max. ∆HU = −28 HU at *ρ* = 0.29 g/cm^3^) and bone (max. ∆HU = 148 HU at *ρ* = 1.93 g/cm^3^). For comparison, Table 1 summarizes the detailed CT numbers for the upright CT and standard of care conventional CT. Figure 3c,d compares body‐ and head‐HLUTs derived from a single session (date corresponding to the anthropomorphic phantom scan) following the consensus configuration for the upright and conventional CT, respectively. For the upright CT, HLUTs showed deviations around adipose/soft tissue (local maximum ∆SPR = 0.072 occurred at −142 HU) and cortical bone (maximum ∆SPR = 0.079 occurred at 1500 HU). For the conventional CT, HLUTs also deviated for cortical bone (maximum ∆SPR = 0.084 occurred at 1500 HU) along with lung (local maximum ∆SPR = 0.020 occurred at −950 HU).

**TABLE 1:** Comparison of CT numbers acquired with the body versus head phantom following the consensus configuration on the upright and conventional CT. For upright CT, mean and standard deviation (SD) of CT numbers are calculated over the 14 timepoints for each insert.

Machine	Upright CT (*N* = 14)			Conventional CT (*N* = 1)		
Metric	Body phantom Mean ± SD (HU)	Head phantom Mean ± SD (HU)	Difference in Mean (HU (%))	Body phantom (HU)	Head phantom (HU)	Difference (HU (%))
Lung LN300	−689 ± 1.1	−681 ± 1.8	8 (1%)	−683	−711	−28 (−4%)
Lung LN450	−514 ± 1.1	−509 ± 0.9	6 (1%)	−521	−535	−14 (−3%)
HE General Adipose	−86 ± 2.0	−56 ± 1.9	30 (35%)	−62	−69	−8 (−12%)
HE Breast 50:50	−52 ± 1.9	−25 ± 2.0	27 (52%)	−37	−37	0 (0%)
Liquid Water	−13 ± 2.1	10 ± 1.7	23 (174%)	1	2	1 (92%)
HE CT Solid Water	−33 ± 2.3	−10 ± 2.0	24 (71%)	5	2	−4 (−67%)
HE Brain	15 ± 2.1	42 ± 1.8	27 (180%)	32	35	3 (9%)
HE Liver	29 ± 2.3	69 ± 1.9	40 (141%)	59	63	3 (6%)
HE Inner Bone	244 ± 2.8	300 ± 2.6	56 (23%)	285	320	35 (12%)
CaCO3 30%	392 ± 3.0	458 ± 3.0	66 (17%)	434	483	49 (11%)
CaCO3 50%	726 ± 3.5	824 ± 4.1	98 (14%)	770	861	91 (12%)
HE Cortical Bone	1213 ± 4.9	1350 ± 6.1	136 (11%)	1261	1409	148 (12%)

*Note*: Wilcoxon signed‐rank test yielded statistically significant differences between quantifications of upright CT numbers using the body versus head phantom for *all* inserts (*p* <0.05).

The impact of beam hardening on CT number was also evaluated based on the location analysis of bone inserts when placed off‐center. On both upright and conventional CT, CT numbers were generally lower when placed in the periphery of the phantom compared with the center location for all four bone inserts with the magnitude of difference slightly higher on the upright CT (∆HU = 9–71 HU, 4%–6% relative to CT number in the center) as compared with the conventional CT (∆HU = 12–21 HU, 2%–4%).

**FIGURE 4:**
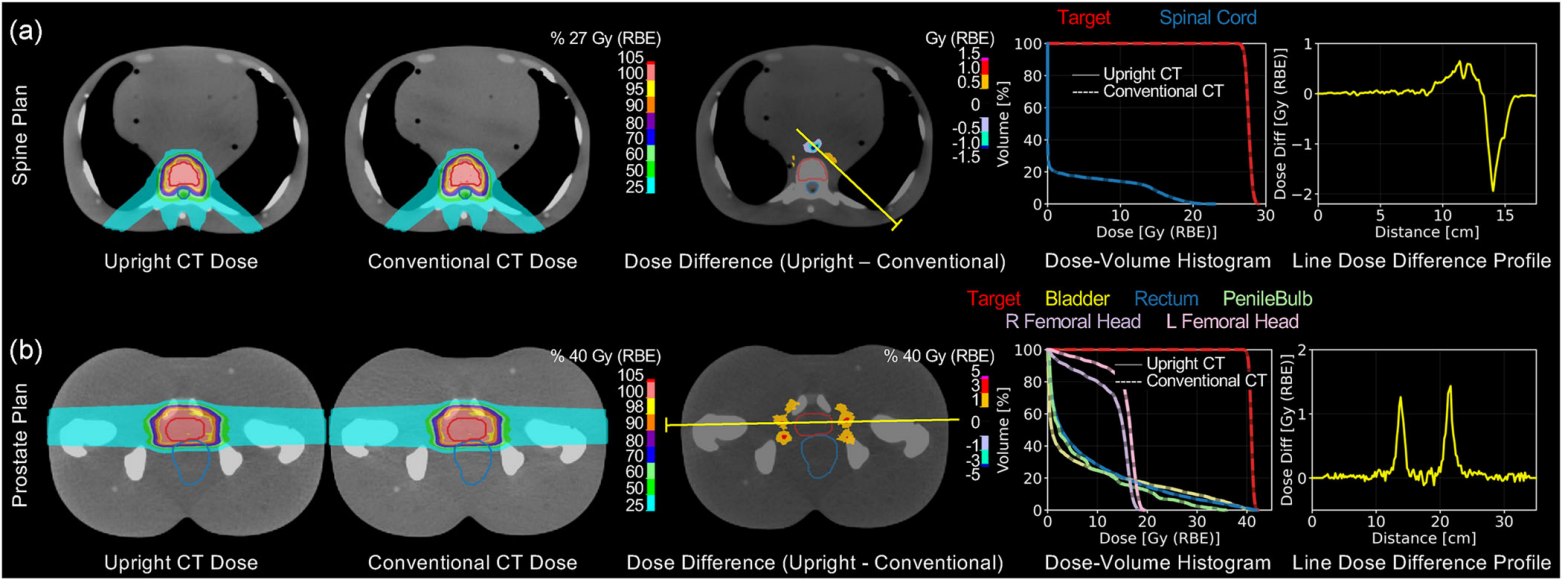
Dosimetric comparison of proton dose calculated on upright versus conventional CT dataset using corresponding Hounsfield look up tables derived following the consensus phantom configuration for representative (a) spine and (b) prostate treatment plans, demonstrating the dose distribution and comparison of dose, dose‐volume histograms, and line dose difference profiles.

Figure 4 demonstrates dosimetric results for an anthropomorphic phantom comparing dose calculated on the upright versus conventional CT datasets using corresponding body HLUTs derived following the consensus configuration for (A) spine and (B) prostate treatment plans. For the spine plan, dose difference maps and line dose difference profiles demonstrated local differences exceeding 1.5 Gy (∼6% of prescription dose) yet with high gamma pass rate γ = 99.8% at 1 mm/1%. Similarly, for the prostate plan, local dose differences greater than 1.2 Gy (3% of prescription dose) on the distal end of proton beam range were present with γ = 99.6%. Clinical DVH curves suggested excellent agreement with minimal target coverage differences (∆D_95 _= −0.3% for the spine plan and ∆D_98 _= −0.1% for the prostate plan).

To quantify the discrepancies of the simplified configuration to the consensus configuration due to increased uncertainties of beam hardening, CT numbers acquired following the consensus versus simplified configuration using the body phantom were compared on both CT scanners as depicted in Supplement Figure S4A,B, with detailed CT numbers summarized in Supplement Table S3. On the upright CT, Wilcoxon signed‐rank test yielded statistically significant differences (*p* <0.05) between CT number quantifications using the consensus versus simplified configuration across all inserts. However, absolute differences in CT numbers from the two configurations were overall small (<18 HU) except for the largest discrepancies in the cortical bone (∆ = −76 HU (−6%)). On the conventional CT, the agreement was excellent and outperformed the upright CT, with the largest absolute difference in the same densest bone insert (∆ = −23 HU (−2%)).

While the simplified configuration is only intended for efficient routine QA and not to derive comprehensive HLUT, an assessment was performed to better understand sensitivity of HLUT specifications to the phantom configuration. Supplement Figure S4C,D compare SPR HLUTs derived from a single session (date corresponding to the anthropomorphic phantom scan) following the consensus versus simplified configuration using the body phantom on the upright and conventional CT, respectively. For the upright CT, HLUTs agreed well except for noticeable deviations around adipose/soft tissue (local maximum ∆SPR = 0.020 occurred at −142 HU) and cortical bone (maximum ∆SPR = 0.044 occurred at 1500 HU). For the conventional CT, HLUTs showed excellent agreement globally with maximum ∆SPR = 0.013 that occurred at 1500 HU.

## 4 DISCUSSION

Our work presents the first longitudinal assessment of upright CT stability over an 8‐month period of operation, which is essential for assessing the reliability of the CT performance for simulation and treatment planning purposes. Data were obtained following consensus guidelines^16^ and using a simplified phantom configuration to evaluate feasibility for establishing a routine QA program. For both consensus and simplified phantom configurations, CT number for the upright CT showed excellent repeatability with the inter‐session variabilities (SD) of the simplified and consensus configurations <5 HU. Similarly, intra‐session variability in the repeat measures of the simplified configuration was ≤1.6 HU. These results were comparable to reported longitudinal performance of a conventional CT where CT number SD = 0.9–6.4 HU for 100 repeat measurements of materials of a Catphan phantom (Phantom Laboratory, Salem, NY) over 6 months,^23^ suggesting equivalent longitudinal stability of upright CT to standard of care conventional CT. These results are meaningful in the context of offline and online adaptive proton therapy, where daily IGRT images may be considered for QACT evaluation. Given that ∼30%–60% head and neck cancer patients and ∼60% thoracic cancer patients are reported to require adaptation in proton therapy,^24^, ^25^, ^26^, ^27^ having the ability to use isocentric, high quality upright CT data for offline replanning in lieu of QACTs is advantageous. Implementing upright CT for routine online or offline ART would enable efficient and reliable dose verification with accurate patient anatomy in the treatment position, thereby eliminating uncertainties from deformable registration which would reduce clinical resources and accelerate the clinical ART workflow.^24^, ^25^

During the 8‐month study, the upright CT underwent software upgrades twice and CT number recalibration once during one of the software upgrades. Systematic CT number offsets (up to ∼9 HU in cortical bone comparing mean CT number) were found after the first software upgrade while undergoing a simultaneous recalibration, yet these results remained consistent after the second upgrade (differences in mean CT number ≤3.2 HU across all inserts compared to measurements after the first upgrade). Because the initial CT number calibration was established in the early development phase of the upright CT, the vendor refined their calibration protocol as their installation base increased. Thus, the recalibration was performed using a more mature service protocol providing better CT number accuracy to expected reference values. Key findings suggest that despite the systematic CT number offset (a decrease of ∼5 HU for most of the inserts), excellent dose agreement was observed between the calculations using different HLUTs (January 9 vs. June 23) with gamma pass rate = 100% at 1 mm/1% for both plans. Nevertheless, following standards for IGRT and CT Simulator QA,^16^, ^28^ post‐upgrade verifications and potential re‐baselining the SPR HLUT are expected after each major system upgrade.

Comparing acquisitions using the body and head phantoms, statistically significant discrepancies (*p* <0.05) were observed with upright CT using the current imaging protocol and system calibrations with the largest difference occurring in the cortical bone with maximum ∆HU = 136 HU (11%) and ∆SPR = 0.079. Discrepancies with similar magnitude were also found on the conventional CT (maximum ∆HU = 148 HU (12%) and ∆SPR = 0.084). These results agree with literature,^29^ suggesting that size‐specific HLUTs would be advantageous to address these size‐dependent discrepancies. On conventional CT, literature has reported to effectively reduce the variations of bone CT number and subsequent SPR measured in different sizes of phantom via applying more advanced iterative beam hardening corrections,^29^ which is currently not available on the upright CT. Efforts could be made to enable iterative beam hardening corrections, further refine the imaging protocols^30^ and establish a CT calibration procedure to improve image quality and reduce the size‐dependent discrepancies.

Compared to standard of care conventional CT, dosimetric evaluation revealed clinically acceptable dosimetric agreement with >99% gamma pass rate and <0.3% difference in target coverage. Future efforts toward improving the dosimetric agreement are warranted such as refining upright CT calibration methods.

One limitation of this work is that the evaluated upright CT system is currently preclinical such that only research activities were being performed over the timeline of the study. For a clinically deployed system that images patients daily, we would expect the system's use and duty cycle to be higher. While limited clinical data exists on daily workloads for upright proton therapy, a mature clinical proton program typically operates on an average of 10 h (up to 16 h) daily.^31^, ^32^ Future longitudinal monitoring of upright CT stability in a clinical environment is warranted to further validate our findings. In addition, the upright CT acquisition and reconstruction protocols are stabilizing but still undergoing improvements. Some residual artifacts can be observed such as the slightly brighter areas in the long‐axis periphery of the oval‐shaped phantom demonstrated in Figure 1d and e, which will be addressed in the future using a new anti‐scatter grid technology. Another limitation of this work is that the experiments were only performed in phantom whereas further validations are warranted using animal tissue^33^, ^34^ and matched‐pair datasets between upright and supine patient scanning that we expect to follow for our clinical patient protocol to assess the accuracy of dose calculation on upright CT and its equivalence toward conventional CT. Future multi‐institutional CT number stability studies are warranted to validate our findings and provide comprehensive frequency recommendations and tolerances for QA tests.

It is important to note that the simplified configuration is not intended for clinical HLUT specification given the observed CT number and SPR discrepancies compared to the established consensus configuration. Instead, it is presented as an approach to consider for routine QA. The faster acquisition using a single scan renders it suitable for more frequent, routine QA to monitor the machine stability, as its longitudinal variability showed minimal differences to scans following the consensus guidelines (simplified vs. consensus intersession SD ≤3.2 HU vs. ≤4.9 HU). While other phantoms such as Catphan^23^ and ACR phantom^35^ may be evaluated for routine QA (e.g., monthly),^28^ these phantoms have limited materials (5–7) that are not suitable tissue surrogates (e.g., polymethylpentene [PMP] ∼−200 HU to Teflon ∼1000 HU) and do not cover the whole physiological range (e.g., lungs< −500 HU and cortical bone >1000 HU). Furthermore, smaller phantoms may not represent appropriate sampling of body‐sized imaging object given the observed size‐dependent discrepancies. A detailed measurement using a body‐sized calibration phantom with tissue‐mimicking inserts that more densely sample physiologically relevant CT numbers may provide a more comprehensive approach to capture critical parameters for future proton online ART while enabling an efficient clinical workflow. Developing a similar simplified configuration for head phantom QA was not pursued in our study because the body phantom is expected to be more susceptible to beam hardening and scattering uncertainties, thus it might be a more sensitive option to monitor CT number changes. Furthermore, the head phantom only holds 10 inserts whereas the body phantom can accommodate all 12 inserts required for HLUT derivation within a single scan.

## 5 CONCLUSION

The upright CT demonstrated excellent longitudinal stability and reliability to support initial CT simulation and future online proton ART implementations. While size‐specific HLUTs are necessary for upright CT, dosimetric evaluation suggested local but clinically insignificant dose discrepancies comparing upright and conventional CT. The simplified configuration demonstrated feasibility to serve as a comprehensive evaluation while balancing efficient QA to monitor machine stability. With further validation across multiple institutions, online upright proton ART can be further established.

## AUTHOR CONTRIBUTIONS

Yuhao Yan contributed to phantom data acquisition, data processing and analysis, and manuscript preparation. Jordan Slagowski contributed to phantom data acquisition, proton planning, and manuscript review. Jessica Miller contributed to data consultation and manuscript review. John Hayes contributed to upright CT development and maintenance, data consultation, and manuscript review. Carson Hoffman contributed to upright CT development and maintenance, data consultation, and manuscript review. Minglei Kang contributed to proton planning and manuscript review. Carri Glide‐Hurst contributed to study conception, manuscript preparation, manuscript review, and overall supervision. All authors approved the final version of the manuscript.

## CONFLICT OF INTEREST STATEMENT

Carri Glide‐Hurst reports research collaborations with RaySearch Laboratories, Leo Cancer Care, Inc., and GE Healthcare (PI: Carri Glide‐Hurst). John Hayes and Carson Hoffman are employees of Leo Cancer Care. Jessica Miller reports research funding from Siemens Healthineers. Yuhao Yan, Jordan Slagowski, and Minglei Kang report no conflicts of interest.

## Supporting information




**Supporting File**: acm270685‐supp‐0001‐SuppMat.docx.

## Data Availability

Research data are not available at this time.
