# Supplementary material for: Longitudinal CT number characterization of a novel upright CT for proton therapy planning
Source: J Appl Clin Med Phys. 2026 Jul 7;27(7):e70685. doi: 10.1002/acm2.70685 (PMC13341640; doi:10.1002/acm2.70685)
Supplement: Supplementary file 1 — Supporting File: acm270685‐supp‐0001‐SuppMat.docx. [file ACM2-27-e70685-s001.docx]

**Longitudinal CT Number Characterization of a Novel Upright CT for Proton Therapy Planning**


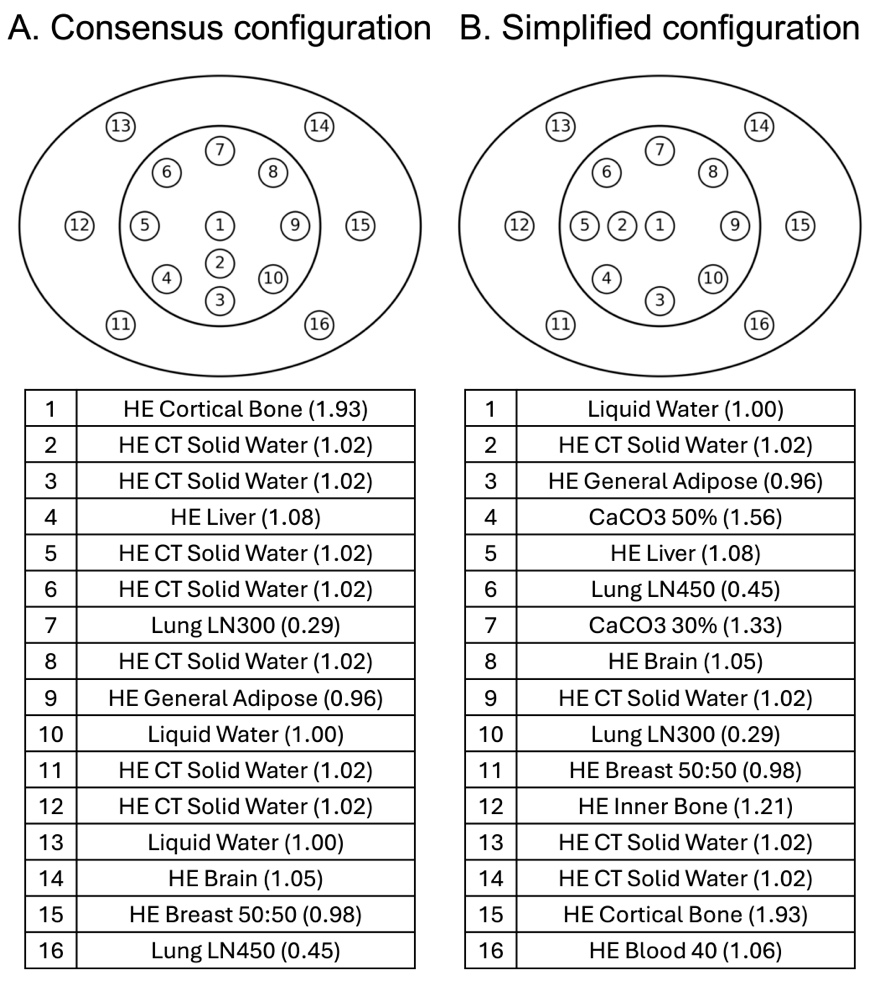


**Figure S1.** Insert indexing for (A) the consensus configuration (Peters *et al*., 2023^1^) and (B) the simplified configuration, corresponding to the phantom photographs and upright CT images in Figure 1. Insert materials and corresponding nominal mass densities in g/cm^3^ are listed in the tables below each schematic.

**Table S1.** Summarized longitudinal (Dec 2024-July 2025) statistics for upright CT numbers of all inserts acquired using different phantom configurations within the body-sized phantom. For intra-session metrics, range was reported. SD: standard deviation. CV: coefficient of variation, in absolute value in case the mean is negative.

| Phantom Configuration | Consensus | | | Simplified | | | | |
| --- | --- | --- | --- | --- | --- | --- | --- | --- |
| Metric | Mean  (HU) | Inter-  session  SD (HU) | Inter-  session  CV  (%) | Mean  (HU) | Intra-session  SD  [min, max] (HU) | Intra-session  CV  [min, max] (%) | Inter-  session  SD (HU) | Inter-  session  CV  (%) |
| Lung LN300 | -689 | 1.1 | 0.2 | -686 | [0.4, 1.3] | [0.1, 0.2] | 0.8 | 0.1 |
| Lung LN450 | -514 | 1.1 | 0.2 | -525 | [0.5, 1.5] | [0.1, 0.3] | 1.2 | 0.2 |
| HE General Adipose | -86 | 2.0 | 2.3 | -88 | [0.5, 1.4] | [0.6, 1.5] | 2.1 | 2.3 |
| HE Breast 50:50 | -52 | 1.9 | 3.7 | -43 | [0.3, 1.2] | [0.7, 2.6] | 1.8 | 4.2 |
| Liquid Water | -13 | 2.1 | 15.6 | -31 | [0.4, 1.3] | [1.3, 4.3] | 2.4 | 7.5 |
| HE CT  Solid Water | -33 | 2.3 | 6.9 | -28 | [0.6, 1.3] | [2.3, 4.7] | 2.0 | 7.1 |
| HE Brain | 15 | 2.1 | 14.1 | 3 | [0.5, 1.3] | [11.8, 1033.3] | 1.8 | 55.0 |
| HE Liver | 29 | 2.3 | 8.1 | 28 | [0.3, 1.4] | [1.1, 5.5] | 1.9 | 6.9 |
| HE Inner Bone | 244 | 2.8 | 1.1 | 233 | [0.3, 1.0] | [0.1, 0.4] | 2.6 | 1.1 |
| CaCO3 30% | 392 | 3.0 | 0.8 | 387 | [0.2, 1.6] | [0.1, 0.4] | 2.4 | 0.6 |
| CaCO3 50% | 726 | 3.5 | 0.5 | 712 | [0.4, 1.3] | [0.1, 0.2] | 3.2 | 0.5 |
| HE Cortical Bone | 1213 | 4.9 | 0.4 | 1137 | [0.2, 1.0] | [0.0, 0.1] | 3.2 | 0.3 |

**Table S2.** Longitudinal upright CT number changes relative to the baseline (the first available timepoint) grouped by system upgrades, acquired following the consensus configuration using the body phantom.

| Timepoint | Baseline | Before 1^st^ upgrade + recalibration (N=7) | | After 1^st^ upgrade + recalibration (N=3) | | After 2^nd^ upgrade (N=3) | |
| --- | --- | --- | --- | --- | --- | --- | --- |
| Metric | CT number (HU) | Mean ∆  (HU (%)) | ∆ range  [lower, upper]  (HU) | Mean ∆  (HU (%)) | ∆ range  [lower, upper]  (HU) | Mean ∆  (HU (%)) | ∆ range  [lower, upper]  (HU) |
| Lung LN300 | -686 | -3.1 (-0.4%) | -4.5, -1.8 | -3.2 (-0.5%) | -3.9, -2.5 | -3.5 (-0.5%) | -3.9, -3.0 |
| Lung LN450 | -513 | -1.3 (-0.2%) | -2.4, -0.7 | -2.8 (-0.5%) | -3.3, -2.2 | -3.1 (-0.6%) | -3.6, -2.5 |
| HE General Adipose | -82 | -3.3 (-4.0%) | -3.9, -2.0 | -6.0 (-7.3%) | -6.8, -5.4 | -6.6 (-8.1%) | -7.1, -6.2 |
| HE Breast 50:50 | -48 | -2.7 (-5.5%) | -4.1, -2.1 | -6.2 (-12.8%) | -7.1, -5.0 | -5.0 (-10.3%) | -6.1, -4.1 |
| Liquid Water | -9 | -2.9 (-31.3%) | -3.8, -2.1 | -7.2 (-77.4%) | -7.5, -6.7 | -4.7 (-50.7%) | -5.7, -4.0 |
| HE CT  Solid Water | -29 | -3.2 (-11.2%) | -4.8, -1.9 | -6.4 (-22.3%) | -7.0, -5.4 | -7.2 (-25.2%) | -8.4, -6.3 |
| HE Brain | 18 | -2.4 (-13.1%) | -3.6, -0.8 | -6.9 (-37.4%) | -7.6, -6.4 | -3.7 (-20.0%) | -4.6, -2.9 |
| HE Liver | 33 | -2.9 (-8.7%) | -4.0, -1.0 | -7.3 (-22.2%) | -7.6, -6.9 | -6.0 (-18.3%) | -6.3, -5.9 |
| HE Inner Bone | 249 | -2.2 (-0.9%) | -3.8, -0.9 | -8.1 (-3.3%) | -8.5, -7.4 | -5.8 (-2.3%) | -6.7, -5.2 |
| CaCO3 30% | 396 | -1.7 (-0.4%) | -2.6, 0.0 | -8.2 (-2.1%) | -8.7, -7.3 | -6.1 (-1.5%) | -7.0, -5.2 |
| CaCO3 50% | 730 | -2.3 (-0.3%) | -3.5, -1.3 | -9.4 (-1.3%) | -9.9, -9.1 | -8.0 (-1.1%) | -9.0, -6.3 |
| HE Cortical Bone | 1221 | -4.3 (-0.4%) | -5.8, -2.7 | -13.6 (-1.1%) | -14.4, -12.5 | -12.9 (-1.1%) | -13.1, -12.5 |

**Additional analysis of the dosimetric impact of longitudinal CT number variations**

During our 8-month study, slight systematic offsets of CT numbers were found after the first software upgrade that occurred with a simultaneous recalibration. Nevertheless, minimal CT number variations were observed with differences <15 HU across all evaluated inserts comparing measurements from any two timepoints. To investigate the dosimetric impact of the observed CT number variations, dose calculations were compared using two HLUTs derived from consensus measurements with all other parameters controlled. Specifically, proton treatment plans were recalculated on the same CT images but with consensus configuration body phantom-derived HLUT from January 9, 2025, the first available timepoint before the first software upgrade with CT number recalibration. Results were compared to those calculated with the HLUT derived from the consensus configuration body phantom from June 23, 2025, the same day as the anthropomorphic phantom scan after the second software upgrade. Spine and prostate treatment plans were recalculated using fixed beam angles, spot positions, spot weights, and monitor units. CT numbers and HLUTs from the two measurements were compared to demonstrate the longitudinal variations, and dose calculations using the two HLUTs were compared to assess the dosimetric impact.

Figure S2 demonstrates the longitudinal variations in CT numbers and resultant differences in HLUTs. As shown in Fig S2A, CT numbers measured on January 9 were systematically higher than June 23 with minor ∆HU ~5 HU for most inserts but 13.1 HU for cortical bone (ρ=1.93 g/cm^3^). Fig S2B demonstrates excellent agreement of the two HLUTs, with Fig S2C suggesting maximum ∆SPR=0.026 occurred at -142 HU.

Figure S3 summarizes comparison of dose calculation using HLUTs from different dates with the same plan and CT image for the spine and prostate plans. For both plans, dose difference maps demonstrated minimal differences. DVH showed excellent agreement with negligible difference in target coverage (∆D_95_=0.0% for the spine plan and ∆D_98_=0.0% for the prostate plan). Gamma test pass rates were 100.0% at 1 mm/1% for both plans.

*
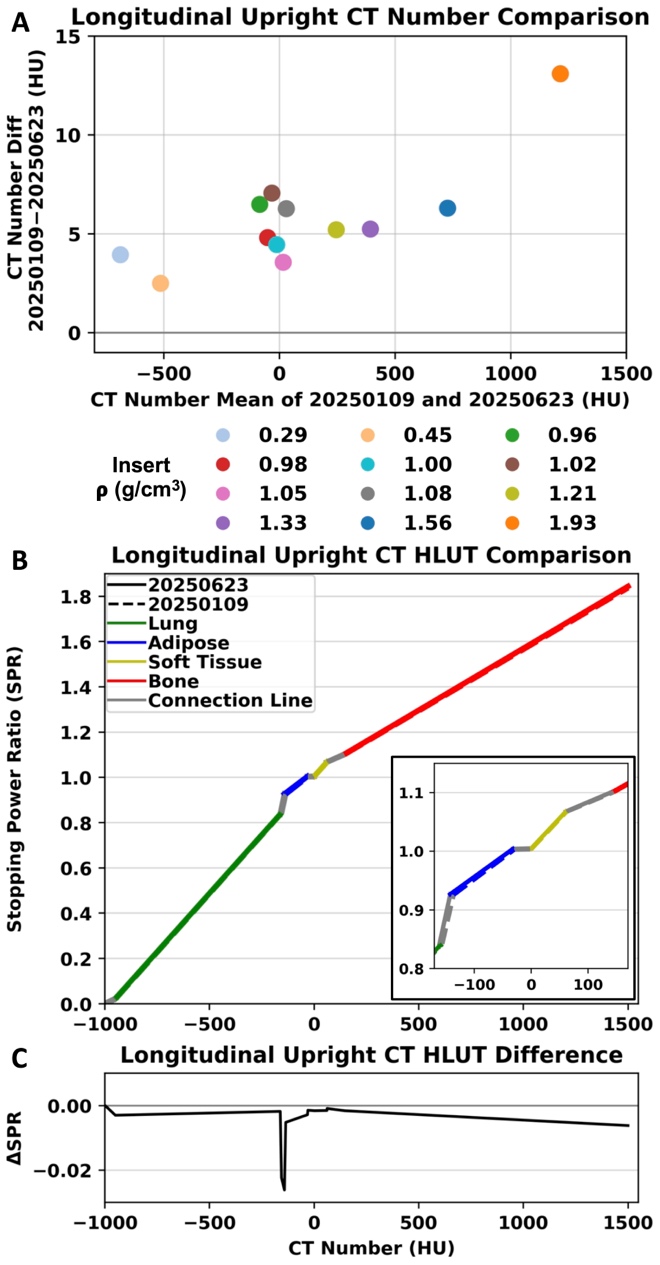
*

**Figure S2.** Comparison of acquisitions on two different dates on upright CT to assess the longitudinal variations in presence of software version and calibration condition changes, including (A) Bland-Altman plot of CT numbers, (B) Hounsfield look-up tables (HLUTs), and (C) HLUT differences. A zoomed-in view highlighting adipose and soft tissue is shown in the bottom right for HLUT. All acquisitions used the body phantom and followed the consensus configuration.


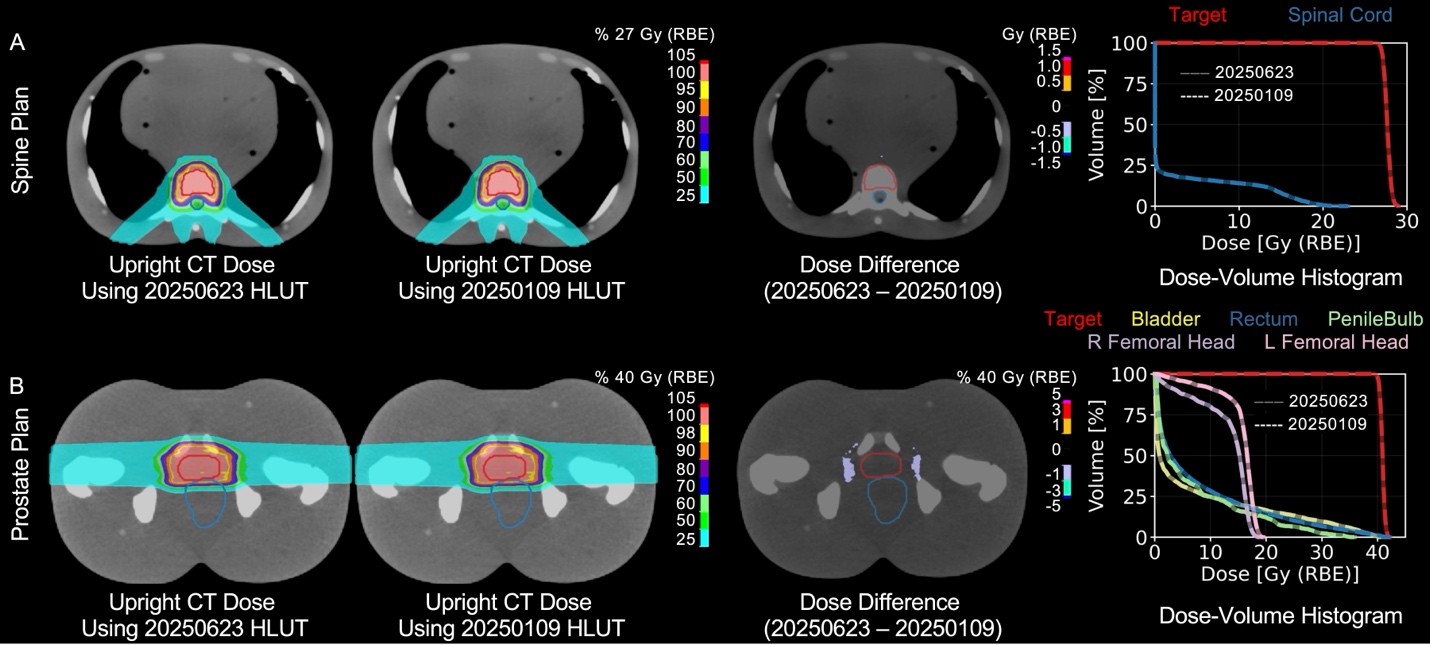


**Figure S3.** Dosimetric comparison of proton dose calculated on the same upright CT dataset acquired on June 23, 2025, using the same plan but different HLUTs derived from consensus measurements on June 23 versus January 9, 2025 for representative (A) spine and (B) prostate treatment plans, demonstrating the dose distribution and comparison of dose and dose-volume histograms.

**Table S3.** Comparison of CT numbers acquired using the consensus versus simplified phantom configuration in the body phantom on the upright and conventional CT. For upright CT, mean and standard deviation (SD) of CT numbers for each insert are calculated over the 14/15 timepoints for consensus/simplified configuration results, respectively.

| Machine | Upright CT | | | Conventional CT | | |
| --- | --- | --- | --- | --- | --- | --- |
| Metric | Consensus  (N=14) Mean±SD (HU) | Simplified  (N=15) Mean±SD (HU) | Difference in Mean  (HU (%)) | Consensus  (N=1)  (HU) | Simplified  (N=1)  (HU) | Difference  (HU (%)) |
| Lung LN300 | -689±1.1 | -686±0.8 | 3 (0%) | -683 | -687 | -4 (-1%) |
| Lung LN450 | -514±1.1 | -525±1.2 | -10 (-2%) | -521 | -513 | 8 (1%) |
| HE General Adipose | -86±2.0 | -88±2.1 | -2 (-2%) | -62 | -61 | 1 (1%) |
| HE Breast 50:50 | -52±1.9 | -43±1.8 | 9 (18%) | -37 | -33 | 4 (10%) |
| Liquid Water | -13±2.1 | -31±2.4 | -18 (-136%) | 1 | 6 | 5 (533%) |
| HE CT Solid Water | -33±2.3 | -28±2.0 | 5 (15%) | 5 | 4 | -2 (-33%) |
| HE Brain | 15±2.1 | 3±1.8 | -12 (-79%) | 32 | 32 | 0 (1%) |
| HE Liver | 29±2.3 | 28±1.9 | -1 (-3%) | 59 | 58 | -1 (-2%) |
| HE Inner Bone | 244±2.8 | 233±2.6 | -11 (-5%) | 285 | 269 | -16 (-5%) |
| CaCO3 30% | 392±3.0 | 387±2.4 | -5 (-1%) | 434 | 430 | -4 (-1%) |
| CaCO3 50% | 726±3.5 | 712±3.2 | -13 (-2%) | 770 | 764 | -6 (-1%) |
| HE Cortical Bone | 1213±4.9 | 1137±3.2 | -76 (-6%) | 1261 | 1237 | -23 (-2%) |
| Wilcoxon signed-rank test yielded statistically significant differences between quantifications of upright CT numbers using the consensus versus simplified configuration for *all* inserts (p<0.05) | | | | | | |


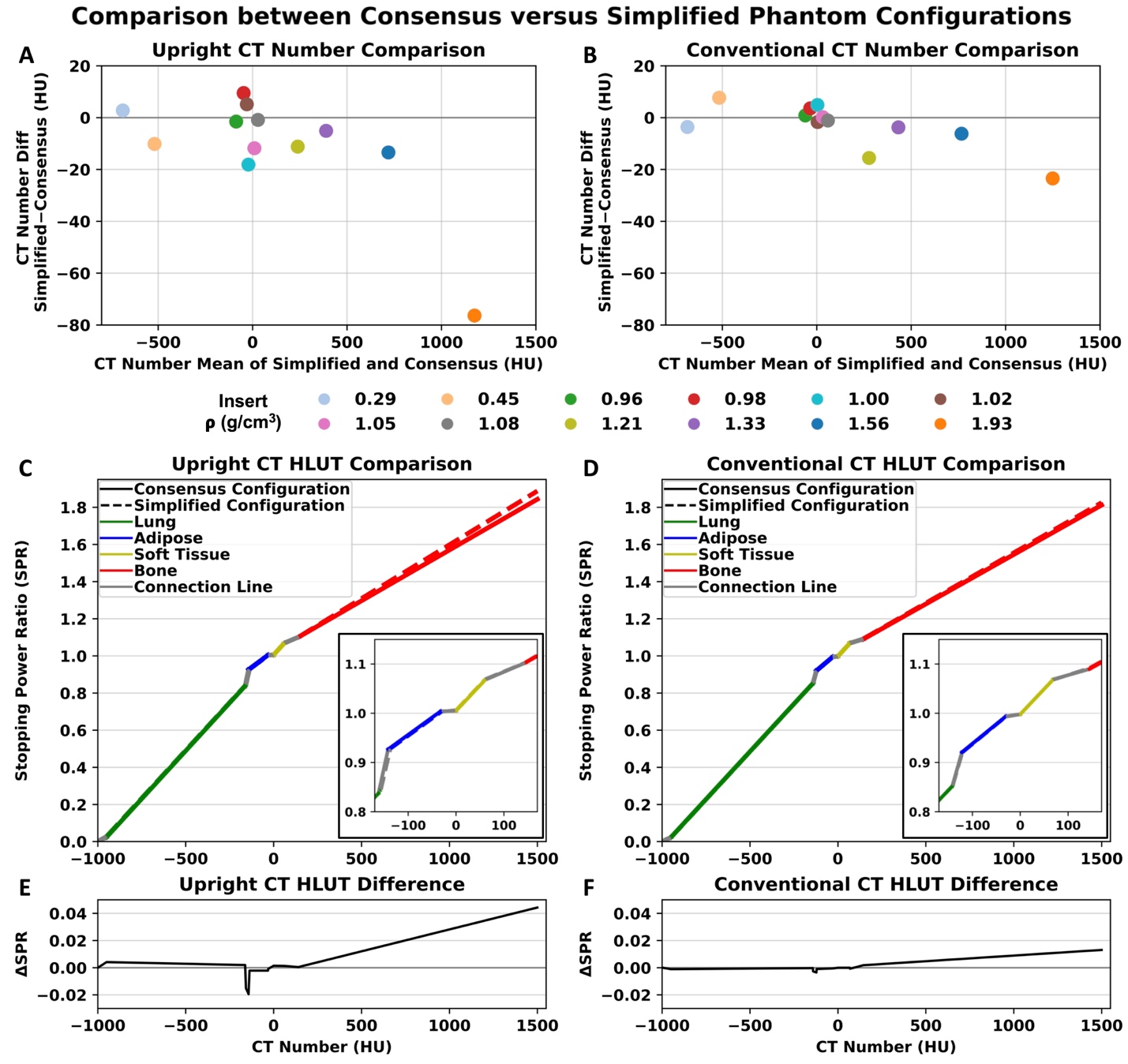


**Figure S4.** Comparison of acquisitions using the consensus versus simplified configuration with the body phantom, including (A) Bland-Altman plot of CT numbers on the upright CT and (B) the conventional CT, and (C) Hounsfield look-up tables (HLUTs) on the upright CT and (D) the conventional CT, and (E) HLUT differences on the upright CT and (F) the conventional CT. A zoomed-in view highlighting adipose and soft tissue is shown in the bottom right for HLUT.

**References**

1. Peters N, Trier Taasti V, Ackermann B, et al. Consensus guide on CT-based prediction of stopping-power ratio using a Hounsfield look-up table for proton therapy. *Radiotherapy and Oncology*. 2023;184:109675. doi:10.1016/J.RADONC.2023.109675
